# Differentially private knowledge transfer for federated learning

**DOI:** 10.1038/s41467-023-38794-x

**Published:** 2023-06-24

**Authors:** Tao Qi, Fangzhao Wu, Chuhan Wu, Liang He, Yongfeng Huang, Xing Xie

**Affiliations:** 1 Department of Electronic Engineering, Tsinghua University, 100084 Beijing, China; 2 Microsoft Research Asia, 100080 Beijing, China; 3 Zhongguancun Laboratory, 100094 Beijing, China; 4 Institute for Precision Medicine, Tsinghua University, 102218 Beijing, China

**Keywords:** Ethics, Engineering

## Abstract

Extracting useful knowledge from big data is important for machine learning. When data is privacy-sensitive and cannot be directly collected, federated learning is a promising option that extracts knowledge from decentralized data by learning and exchanging model parameters, rather than raw data. However, model parameters may encode not only non-private knowledge but also private information of local data, thereby transferring knowledge via model parameters is not privacy-secure. Here, we present a knowledge transfer method named PrivateKT, which uses actively selected small public data to transfer high-quality knowledge in federated learning with privacy guarantees. We verify PrivateKT on three different datasets, and results show that PrivateKT can maximally reduce 84% of the performance gap between centralized learning and existing federated learning methods under strict differential privacy restrictions. PrivateKT provides a potential direction to effective and privacy-preserving knowledge transfer in machine intelligent systems.

## Abstract (web summary)

To ensure the privacy of processed data, federated learning approaches involve local differential privacy techniques which however require communicating a large amount of data that needs protection. The authors propose here a framework that uses selected small data to transfer knowledge in federated learning with privacy guarantees.

## Introduction

In recent years, machine learning technology has developed rapidly and empowered intelligent systems in many real-world scenarios, such as intelligent healthcare^1–3^ and social computing^4–7^. The success of machine learning usually lies in summarizing useful knowledge from big data, which is mainly benefited from the high capacity and complexity of models^8–10^. Learning machine learning models on centralized data is a mainstream knowledge transfer paradigm^11^. However, training data in many tasks are highly privacy-sensitive^12,13^, and recent privacy leakage accidents have drawn more and more attention from the public to the security of user privacy^14–16^. Moreover, some strict privacy regulations such as GDPR^17^ and CCPA^18^ are also spawned to limit the collection, processing, and storage of user data^19–21^. Due to the privacy-sensitive nature of training data, the centralized model training usually arouses serious privacy concerns and even violates privacy regulations^22–24^.

**Fig. 1:**
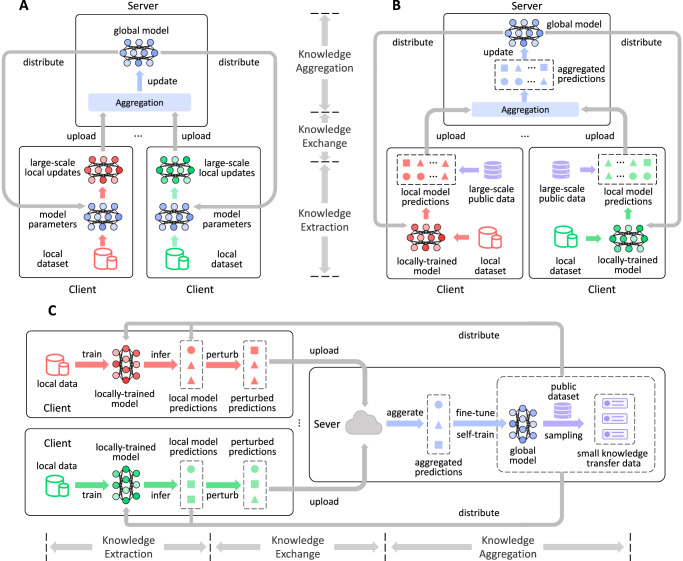
The differences between our work and prior methods. **A** The federated learning method that transfers knowledge via local model updates. **B** The federated learning method that transfers knowledge via local model predictions on a large-scale unlabeled public dataset. **C** The proposed PrivateKT method that transfers knowledge via actively selected small public data.

Federated learning (FL) can transfer knowledge from decentralized data^8,25,26^, and thereby begins to serve as a privacy-aware model training framework in many privacy-sensitive applications, such as Covid-19 patient detection^27,28^ and intelligent personal assistant^29,30^. In federated learning, knowledge is usually extracted from decentralized data into local model updates, and further aggregated into a shared model by communicating local model updates rather than raw data (Fig. 1a)^8,24,31–33^. However, model updates usually have enough capacity to memorize the private information in training data, the disclosure of which still has the risks of leaking raw data^34,35^. To improve privacy security, some federated learning methods propose to transfer knowledge based on a large-scale unlabeled public dataset (Fig. 1b)^36–38^. In these methods, each client first extracts knowledge from decentralized data into local predictions on the entire unlabeled public dataset, then the server aggregates the uploaded local predictions to update a global model. However, the local model predictions are also correlated to private information in local data, and thereby user privacy is still not guaranteed in these works^39^.

To protect user privacy in federated learning, local differential privacy (LDP) that perturbs communicated data with noise can be applied to offer theoretical privacy guarantees^40^. Nevertheless, the intensity of LDP noise is usually proportional to the size of communicated data^41^, making it ineffective for LDP to protect existing federated learning methods that depend on large-scale public data for knowledge transfer. A naive solution is to reduce the size of public data, however, knowledge transfer via small data is usually ineffective and may seriously degrade the model performance^42^. Thus, in this work, we study how to effectively and privately transfer knowledge via a small amount of data. The core idea of our solution is that, the server actively selects a small amount of public data informative for model learning as the carrier of knowledge transfer (Fig. 1c; named as PrivateKT). In order to achieve privacy guarantees, PrivateKT locally perturbs predictions of local models on the carefully selected data via randomized response mechanism^43^. Then the server in PrivateKT collects and aggregates the local knowledge to update the model. We evaluate PrivateKT on three benchmark datasets for three different real-world tasks. Extensive experiments show that, under strict privacy guarantees, many existing federated learning methods almost degrade into random guesses while PrivateKT can achieve comparable performance with centralized learning (1% loss minimally). Through extensive analysis, we also reveal that small carefully selected data has enough information capacity to transfer big knowledge, which can inspire researchers to design private, effective, and efficient knowledge transfer systems in the future.

## Results

### Overall framework

Next, we first briefly introduce the overall framework of PrivateKT for private knowledge transfer (Fig. 1c). It can extract the local knowledge from decentralized data to collaboratively learn an intelligent model under differential privacy guarantees. In PrivateKT, training data is locally kept by different clients and never shared with the outside, and an intelligent model is maintained by a central server and has a local copy on each client. Besides, following previous works^36,37^, we assume that there is an unlabeled public dataset that is non-privacy sensitive and can be shared across different parties for knowledge transfer.

The knowledge transfer in PrivateKT includes three steps, namely, knowledge extraction, knowledge exchange, and knowledge aggregation. In the knowledge extraction step, each client first trains the intelligent model on its local data and then computes model predictions on a small amount of knowledge transfer data (named KT data). The KT data is actively sampled from the public dataset by the server based on an importance sampling mechanism, where the public data with lower model confidence will be assigned a higher sampling opportunity. In the knowledge exchange step, we share the local knowledge with the server under differential privacy guarantees. Each client locally perturbs model predictions via the randomized response mechanism^43^, which randomly chooses whether to replace a local model prediction with a randomly-generated category label before sending it to the server. In the knowledge aggregation step, the server aggregates uploaded perturbed local predictions and stores them in a knowledge buffer. In this way, the historical aggregated knowledge in the past several rounds is kept by the knowledge buffer, and can be further encoded into the global model by fine-tuning the global model on the knowledge buffer. Moreover, we employ a self-training method^44^ to update the global model on the unlabeled public data to accelerate the model convergence. By repeating this process, we can privately transfer high-quality knowledge from decentralized data into the shared intelligent model.

### Performance evaluation

We conduct experiments in three real-world tasks for performance evaluation. The first task is handwritten digit classification, which needs to classify the category of a digit written by a user. It is based on a widely used federated learning benchmark dataset (named MNIST)^45^. The second task is text-based disease prediction, which needs to predict the diseases described in medical abstracts. It is based on a public dataset (named MedText) released by a Kaggle medical text mining competition. The third task is image-based pneumonia detection, which aims to detect pneumonia from chest X-ray images. This task is based on a real-world dataset (named X-ray) released in a Kaggle X-Ray analysis competition. Following the previous work^36^, 20% of training data is used as the candidate unlabeled data pool to choose samples for knowledge transfer. The model performance is compared under both independent identical data distribution (IID) and non-identical independent data distribution (Non-IID). The non-IID data distributions used for evaluation in experiments include the class non-IID distribution where data classes of local clients are imbalanced, the size non-IID distribution where data sizes of local clients are imbalanced, and the mixed non-IID distribution where both data classes and data sizes of local clients are imbalanced. The IID and Non-IID data partition strategies follow the settings of previous work^46^. (We show more details of datasets in the Supplementary Information.)

The architecture of the basic models trained on MNIST and X-Ray is a two-layer convolution network^45^, and the basic model trained on MedText is a transformer network^47^. Several representative knowledge transfer methods for federated learning methods are compared in experiments, including FedSGD^8^, FedAvg^8^, FedAdam^31^, FedMD^37^, and FedED^36^. The comparison includes results of using LDP or not. We apply LDP to protect the baseline federated learning methods by adding noise to the exchanged local model updates or model predictions. We use the definition of *ϵ*-LDP and the privacy budget *ϵ* is set to 5 (see the “Methods” section). We also include the results of centralized model training (named CenTrain) as a reference for performance comparisons. We use the accuracy as the metric for evaluation on MNIST, and the Macro-F1 as the metric for evaluation on MedText and X-Ray. (More detailed experiential settings are presented in the Supplementary Information.)

**Fig. 2:**
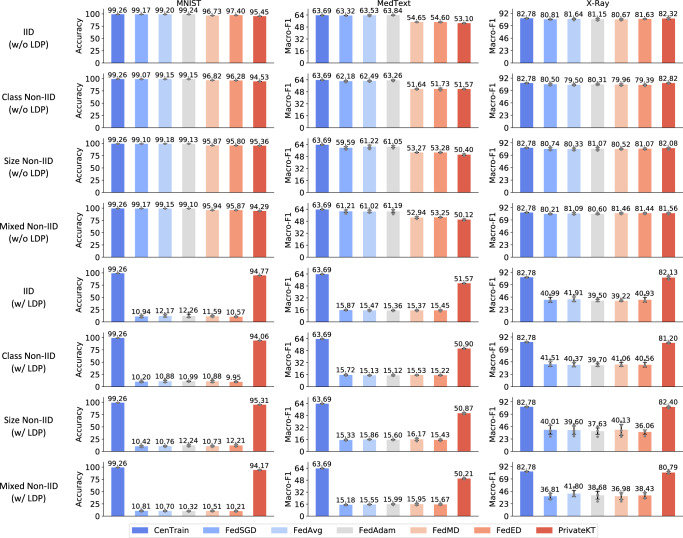
Model performance of different methods on three datasets. The error bars represent the mean results with standard deviations. The comparisons are based on different data distributions and using LDP or not (*ϵ* = 5). The data distributions includes the IID data distribution, imbalanced data class distribution (Class Non-IID), imbalanced data size distribution (Size Non-IID), and distribution where both local data sizes and classes are imbalanced (Mixed Non-IID). The averaged performance and corresponding standard deviations are shown. The results show that the LDP noise seriously hurts the performance of existing federated methods, and PrivateKT can significantly improve the model performance under the same privacy guarantees (*p* < 1*e* − 4 based on *t*-test).

We independently repeat each experiment five times and report the average performance with standard deviations (Fig. 2). From the results, we find that without differential privacy both PrivateKT and other federated learning methods achieve comparable performance with centralized model training. However, when we apply LDP to protect user privacy, the LDP noise seriously hurts the performance of existing federated learning methods. For example, FedAvg drops 86.85% of accuracy on MNIST under the IID data distribution, and FedMD drops 41.91% of Macro-F1 on X-Ray under the class non-IID data distribution. This is because these federated learning methods transfer knowledge by exchanging a large volume of intermediate variables (such as local model updates). However, the intensity of LDP noise is usually proportional to the size of communicated data, making it ineffective for these methods to balance knowledge utility and privacy protection. By contrast, PrivateKT effectively improves the performance of federated learning under the same differential privacy guarantees, and significantly outperforms previous methods (*p* < 1*e* − 4 based on *t*-test). This is because PrivateKT uses small carefully selected data to condense high-quality knowledge, which does not substantially suffer from the perturbation of LDP noise meanwhile improving the effectiveness of knowledge transfer. We then analyze the contributions of different mechanisms in PrivateKT in the following section. We also compare the efficiency and generality of PrivateKT with other FL methods in Supplementary Information. It is worth noting that the evaluation presented in Fig. 2 is primarily founded on moderately large datasets with thousands or tens of thousands of samples (e.g., MNIST and X-Ray). Therefore, further exploration is necessary to fully assess the effectiveness of PrivateKT on large-scale datasets. In light of this, we undertake further analysis by comparing various methods on two larger FL benchmark datasets, CIFAR-10 and CIFAR-100^48^, which are expounded upon in the Supplementary Information. Results show that the main conclusions of our paper still hold: PrivateKT effectively improves the performance of other FL methods under strict privacy restrictions.

### Model effectiveness

**Fig. 3:**
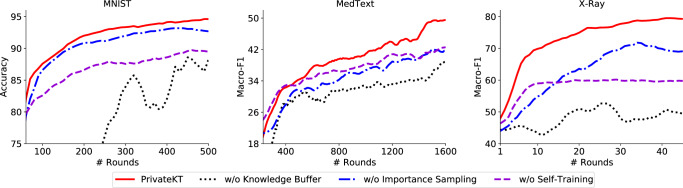
The impacts of important mechanisms in PrivateKT on knowledge transfer. We analyze the impacts of the knowledge buffer and the self-training mechanisms by removing them from PrivateKT individually, and the impact of the importance sampling mechanism by replacing it with a uniform sampling method. Results show that all of these mechanisms can enhance knowledge transfer in PrivateKT, and the knowledge buffer makes the greatest contribution.

Next, we verify the impacts of several important mechanisms in PrivateKT on knowledge transfer, i.e., knowledge buffer, importance sampling, and self-training (Fig. 3). We remove the knowledge buffer and the self-training method from PrivateKT, and replace the importance sampling mechanism with a uniform sampling method, individually, to verify their effectiveness. Results show that both the knowledge buffer and the importance sampling mechanisms effectively improve the model performance and accelerate the model convergence. This is because, in order to mitigate the damage of LDP noise on model performance, PrivateKT only uses a small amount of data sampled from an unlabeled public dataset for knowledge transfer. However, small public data may be insufficient for the effective knowledge transfer and result in suboptimal model performance. Thus, in PrivateKT we tackle this challenge from two aspects. First, we propose to sample knowledge transfer data based on their informativeness for model training, to maximize the quality of knowledge carried by the small sampled data. Second, we propose a knowledge buffer to store and encode historical useful knowledge to the global model, aiming to incorporate more useful knowledge for model updating. Moreover, to enhance the knowledge transfer in PrivateKT, we also employ the self-training technique to further fine-tune the global model, whose contribution is also verified by the results. These results show that PrivateKT can exploit small data for transferring big knowledge. This finding reveals that, big knowledge is not necessarily obtained from big data, but also can be mined from small but representative data.

**Fig. 4:**
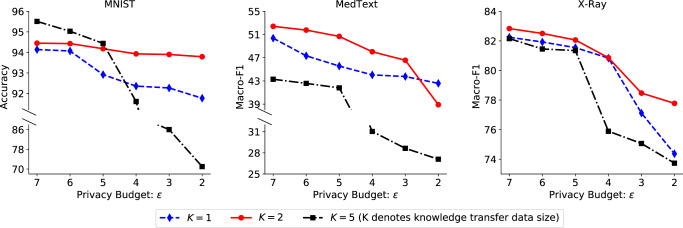
Analysis on the utility-privacy tradeoff in PrivateKT. The model performance under various privacy budgets (i.e., *ϵ*) and sizes of knowledge transfer samples (i.e., *K*) are presented in this figure. The results show that PrivateKT can achieve satisfactory performance under very strong privacy guarantees (e.g., *ϵ* = 2).

Next, we analyze the trade-off between the performance and privacy of the knowledge transfer in PrivateKT (Fig. 4). We show the performance of PrivateKT under different privacy budgets (denoted as *ϵ*) and different knowledge transfer sample sizes (denoted as *K*), where a smaller privacy budget means a stronger differential privacy guarantee. We find that strong differential privacy guarantees do not seriously hurt the model accuracy. For example, the best accuracy of PrivateKT on MNIST is around 94% under a strong privacy guarantee, i.e., *ϵ* = 2. The results verify that PrivateKT can effectively balance the knowledge utility and privacy protection in federated learning. We also find that in each round of PrivateKT a small number of samples (e.g., 2) are sufficient for privately and effectively extracting knowledge from decentralized data to a shared model, which further confirms that, small data is possible for transferring big knowledge. More detailed analyses on the hyper-parameter settings of PrivateKT are shown in Supplementary Information.

## Discussion

From big data to big knowledge, is an important vision of the current machine learning research^8,10^. Federated learning that transfers knowledge from decentralized data into a shared intelligent model is widely used to reduce user privacy risks during knowledge transfer^8,23^. Nevertheless, its privacy security is not guaranteed and needs to be protected by some privacy protection methods such as local differential privacy^40^. However, in this work, we discover that previous federated learning methods are less performant in trading off privacy protection and knowledge utility. Though simply reducing the size of exchanged data is a potential solution to this problem, it may substantially degrade the knowledge transfer effectiveness and thereby yield suboptimal model accuracy^42^. Thus, there raises a question that whether it is possible to transfer big knowledge via small data with strong privacy guarantees.

In this work, we reveal that the answer to the above question is true. The core of achieving this goal is selecting public samples based on their informativeness rather than randomly. After knowledge extraction through these representative samples on local clients, high-quality knowledge encoded by local models can be transferred to the server for aggregation to prepare for the further round of updates. This paradigm enables privacy-preserving knowledge transfer on small data that can minimally suffer from the performance degradation brought by differential privacy, which shows a novel direction to train machine learning models on decentralized data to exploit swarm intelligence. It can also attract further attention to more sophisticated data exploitation methods, rather than simply collecting and involving more and more training data, which is not beneficial for understanding the bound of machine intelligence under limited real-world data and reducing the environmental pollution brought by the computation. We hope our work can further inspire researchers to facilitate knowledge engineering in a more effective, efficient, and privacy-preserving way.

However, our work also has the following limitations. First, the private knowledge transfer method in PrivateKT requires an unlabeled dataset that can be shared across different parties, which may be inaccessible in some applications (e.g., personalized e-commerce). Fortunately, some latest research works find that the knowledge transfer can be effectively performed in a data-free manner^49–51^. Thus, we plan to apply the data-free knowledge transfer methods to PrivateKT to improve its practicability in real-world scenarios. Second, in real-world applications, PrivateKT has the risk of being attacked by Byzantine clients^52^. Therefore, in our future work, we plan to study how to defend the attack to improve the robustness of PrivateKT based on previous works^52–54^. Third, compared with the privacy-invasive centralized training, PrivateKT faces more significant performance degradation on larger datasets. This is because, according to the no free lunch theorem for privacy security and algorithm utility of federated learning^55^, stronger privacy protection will lead to poorer algorithm performance. Since preserving the privacy of a larger volume of training data usually needs stronger protection, the performance degradation of PrivateKT will also become more serious. Thus, in our future work, we will explore improving the privacy-preserving knowledge transfer mechanism of PrivateKT to approach the theoretical performance upper bound. Besides, the demonstration of PrivateKT is mainly based on moderately large datasets (e.g., MNIST and CIFAR), and we admit that the superiority of PrivateKT over other FL methods is not guaranteed on large-scale datasets. In practice, we think our approach is applicable in most scenarios with small or moderate data volumes, and the scalability on huge datasets needs further exploration.

## Methods

Next, we will present the differentially private knowledge transfer method for federated learning (named PrivateKT). We will first give former definitions of local differential privacy and the research problem studied in this paper, and then introduce the details of our PrivateKT method.

### Preliminary

The local differential privacy method (LDP)^56^ aims to protect user privacy under theoretical guarantees. The core idea of LDP is to perturb the shared data via a randomized mechanism to guarantee privacy security. Formally, the definition of LDP can be summarized as follow: a randomized mechanism ${{{{{{{\mathcal{M}}}}}}}}$( ⋅ ) can protect the input data ⋅ under *ϵ*-LDP, if and only if for two arbitrary input data *X* and ${X}^{{\prime} }$, and any output $Y\in range({{{{{{{\mathcal{M}}}}}}}})$, the following inequation holds:

1 $$Pr[{{{{{{{\mathcal{M}}}}}}}}(X)=Y]\le {e}^{\epsilon }\cdot Pr[{{{{{{{\mathcal{M}}}}}}}}({X}^{{\prime} })=Y],$$

where *Pr*[⋅] is the probability of ⋅ , and *ϵ* is the privacy budget. The privacy budget *ϵ* quantifies the privacy guarantee, where a smaller privacy budget means stronger privacy protection.

### Problem definition

Following popular federated learning settings, PrivateKT includes *N* clients and a central server. Each client privately keeps its local dataset and never shares it with the outside, where the local dataset in the *i*-th client is denoted as ${{{{{{{{\mathcal{D}}}}}}}}}_{l}^{i}$. The global model is maintained by the central server and has a local copy on each client. The central server is also responsible for coordinating the clients to participate in the knowledge transfer. In addition, we assume that there is an unlabeled public dataset ${{{{{{{{\mathcal{D}}}}}}}}}_{p}$ that is non-privacy sensitive and can be shared across different parities for knowledge transfer, where the *i*-th sample in ${{{{{{{{\mathcal{D}}}}}}}}}_{p}$ is denoted as ${x}_{i}^{p}$. In order to guarantee privacy security during knowledge transfer, any communicated variables correlated to the local private data need to be protected by the LDP method. The research problem studied in this paper is to design a both private and effective knowledge transfer method for federated learning.

### Differential private knowledge transfer

**Fig. 5:**
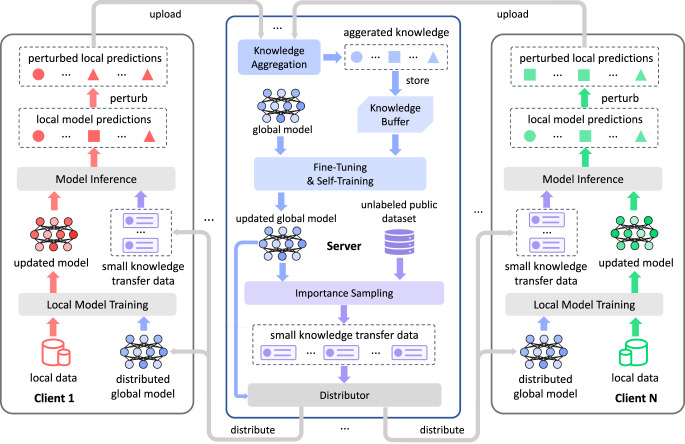
The framework of our PrivateKT method.

The core of private knowledge transfer is communicating perturbed local model predictions on a small amount of actively selected public data. By drastically reducing the size of communicated variables, PrivateKT can effectively mitigate the damage of LDP noise on model performance. Nevertheless, randomly sampled small data may be insufficient to transfer high-quality knowledge from local data to a global model. Thus, we further propose several mechanisms to improve the effectiveness of knowledge transfer based on small data. Next, we will introduce the details of the differential private knowledge transfer in PrivateKT (Fig. 5).

Take the *t*-th knowledge transfer round as an example, PrivateKT includes three core steps, i.e., knowledge extraction, knowledge exchange, and knowledge aggregation. The knowledge extraction step aims to extract knowledge from local data and encode it into local predictions on small actively sampled data. Specifically, the server first distributes the global model in the *t*-round (denoted as Θ_*t*_) and *K* pieces of knowledge transfer (KT) data to each client, and selects a part of clients for model training, where the selected client set is denoted as ${{{{{{{{\mathcal{G}}}}}}}}}_{t}$. (The sampling mechanism of KT data will be introduced in the next paragraph.) For an arbitrary client $c\in {{{{{{{{\mathcal{G}}}}}}}}}_{t}$, it first trains the latest model Θ_*t*_ on its local dataset ${{{{{{{{\mathcal{D}}}}}}}}}_{l}^{c}$. Then the client *c* computes predictions of the locally-trained model on the KT data for knowledge extraction, where ${x}_{i}^{t}$ denotes the *i*-th KT data and ${y}_{c,i}^{t}$ denotes the local model prediction of the client *c* on ${x}_{i}^{t}$. In this way, knowledge can be extracted from local data into local model predictions, and exchanging local predictions can transfer local knowledge to the central server.

However, the local model predictions are correlated to the private data, the disclosure of which remains the risk of leaking raw data. Thus, to guarantee user privacy security under LDP, each client locally perturbs local predictions via the randomized response mechanism^43^. Specifically, for each local model prediction **y**, each client *c* randomly chooses whether replace it with a randomly-generated category label **f** before uploading it to the server:

2 $$\hat{{{{{{{{\bf{y}}}}}}}}}=\left\{\begin{array}{r}{{{{{{{\bf{y}}}}}}}},\quad R=1\\ {{{{{{{\bf{f}}}}}}}},\quad R=0\end{array}\right.,\quad R \sim {{{{{{{\mathcal{B}}}}}}}}(\beta ),\quad {{{{{{{\bf{f}}}}}}}} \sim {{{{{{{\mathcal{P}}}}}}}}(C),$$

where **y** ∈ {0, 1}^*C*^ is the one-hot category vector predicted by the local model, **f** ∈ {0, 1}^*C*^ is a random one-hot vector drawn from a uniform multinomial distribution ${{{{{{{\mathcal{P}}}}}}}}(C)$, $\hat{{{{{{{{\bf{y}}}}}}}}}$ is the perturbed local prediction, *R* is a random variable drawn from a Bernoulli distribution ${{{{{{{\mathcal{B}}}}}}}}(\beta )$, *C* is the number of classification categories and *β* is the probability of assigning the Bernoulli random variable *R* to 1. Based on the randomized response mechanism, the client *c* can build the perturbed local predictions $\{{\hat{{{{{{{{\bf{y}}}}}}}}}}_{c,i}^{t}|i=1,2,...,K\}$ for the knowledge transfer data. By uploading the perturbed predictions to the server, we can privately exchange local knowledge under differential privacy guarantees. (Discussions on privacy guarantees are in the next section.)

After the server collects perturbed predictions from selected clients ${{{{{{{{\mathcal{G}}}}}}}}}_{t}$, the knowledge aggregation step can be executed to update the global model. The sever first aggregates the local predictions on the same KT data to estimate the averaged predictions of different local models on it. Take the *i*-th knowledge transfer data ${x}_{i}^{t}$ as an example, the averaged prediction ${{{{{{{{\bf{y}}}}}}}}}_{i}^{t}=\frac{1}{|{{{{{{{{\mathcal{G}}}}}}}}}_{t}|}{\sum }_{c\in {{{{{{{{\mathcal{G}}}}}}}}}_{t}}{{{{{{{{\bf{y}}}}}}}}}_{c,i}^{t}$ on ${x}_{i}^{t}$ is estimated based on the following equation:

3 $${\hat{{{{{{{{\bf{y}}}}}}}}}}_{i}^{t}=\frac{1}{\beta }\left(\frac{1}{|{{{{{{{{\mathcal{G}}}}}}}}}_{t}|}\mathop{\sum}\limits_{c\in {{{{{{{{\mathcal{G}}}}}}}}}_{t}}{\hat{{{{{{{{\bf{y}}}}}}}}}}_{c,i}^{t}-\frac{1-\beta }{C}{{{{{{{\bf{1}}}}}}}}\right),$$

where ${\hat{{{{{{{{\bf{y}}}}}}}}}}_{i}^{t}$ is an unbiased estimation of ${{{{{{{{\bf{y}}}}}}}}}_{i}^{t}$ and the mean square error of the estimation can asymptotically converge to 0. (The proof is in Supplementary Information.) In this way, the LDP noise can be reduced in the aggregated knowledge, and fine-tuning the global model on the aggregated knowledge can effectively mitigate the damage of LDP noise on knowledge transfer.

Recall that, due to the proportional relation between the LDP noise intensity and communicated data volume, in PrivateKT only a small amount of public data is used for knowledge transfer to mitigate the damage of LDP noise. However, small data may be insufficient to serve as a high-quality carrier to transfer knowledge, which may lead to a suboptimal model performance. To tackle this challenge, we propose two mechanisms to enhance knowledge transfer from different aspects. First, we propose an importance sampling mechanism to maximize the knowledge capacity of KT data for training the global model Θ^*t*^. In this mechanism, we measure the uncertainty of the global model Θ^*t*^ on each unlabeled data in ${{{{{{{{\mathcal{D}}}}}}}}}_{p}$ based on the information entropy, and assign a higher sampling opportunity to unlabeled data with higher model uncertainty. The model uncertainty ${u}_{i}^{d}$ and the sampling weight ${w}_{i}^{d}$ of the *i*-th unlabeled data ${x}_{i}^{p}$ in ${{{{{{{{\mathcal{D}}}}}}}}}_{p}$ are computed as follow:

4 $${w}_{i}^{d}=\frac{\exp ({u}_{i}^{d})}{\mathop{\sum }\nolimits_{j=1}^{|{{{{{{{{\mathcal{D}}}}}}}}}_{p}|}\exp ({u}_{j}^{d})},\quad {u}_{i}^{d}=-\mathop{\sum }\limits_{j=1}^{C}p({x}_{i}^{p},j;{{{\Theta }}}_{t})\log p({x}_{i}^{p},j;{{{\Theta }}}_{t}),$$

where $p({x}_{i}^{p},j;{{{\Theta }}}_{t})$ is the probability of classifying ${x}_{i}^{p}$ to the *j*-th category based on model Θ_*t*_. Second, we propose a knowledge buffer to store historical aggregated knowledge, aiming to encode more useful knowledge to the global model. The server first stores the aggregated knowledge of the current round in the knowledge buffer and then utilizes the knowledge in the buffer to fine-tune the global model Θ_*t*_. (The updated global model is denoted as ${{{\Theta }}}_{t}^{{\prime} }$.) The knowledge buffer is of size *B* and maintains the stored knowledge in a first-in-first-out manner.

Moreover, to accelerate the model convergence, we employ the self-training technique^44^ to further fine-tune the global model ${{{\Theta }}}_{t}^{{\prime} }$. We randomly select *M* samples with low model uncertainties from ${{{{{{{{\mathcal{D}}}}}}}}}_{p}$ and utilize them to self-train the model ${{{\Theta }}}_{t}^{{\prime} }$:

5 $${w}_{i}^{s}=\frac{\exp (-{u}_{i}^{s})}{\mathop{\sum }\nolimits_{j=1}^{|{{{{{{{{\mathcal{D}}}}}}}}}_{p}|}\exp (-{u}_{j}^{s})},\quad {u}_{i}^{s}=-\mathop{\sum }\limits_{j=1}^{C}p({x}_{i}^{p},j;{{{\Theta }}}_{t}^{{\prime} })\log p({x}_{i}^{p},j;{{{\Theta }}}_{t}^{{\prime} }),$$

where ${u}_{i}^{s}$ is the uncertainty of model ${{{\Theta }}}_{t}^{{\prime} }$ on ${x}_{i}^{p}$ and ${w}_{i}^{s}$ is the sampling opportunity of ${x}_{i}^{p}$ for the self-training. Until now, we have finished a knowledge transfer round in PrivateKT and privately transferred knowledge from decentralized data to the global model, where the updated model is denoted as Θ^*t*+1^. Furthermore, we can continue the next knowledge transfer round, after the server distributes the latest global model Θ^*t*+1^ and corresponding KT data to local clients. By repeating this process, we can transfer knowledge from decentralized data to collaboratively learn an intelligent model in an effective and privacy-preserving way. The workflow of PrivateKT is also summarized in Algorithm 1.

### Algorithm pseudo code

#### Algorithm 1

**Workflow of PrivateKT**

1: Setting the hyperparameters *ϵ*, *K*, *β*, *B*, *M* and *T*

2: Sever randomly initializes the model parameter Θ_1_

3: Server randomly selects *K* knowledge transfer data ${{{{{{{{\mathcal{D}}}}}}}}}_{d}^{1}=\{{x}_{i}^{1}|i=1,...,K\}$ from ${{{{{{{{\mathcal{D}}}}}}}}}_{p}$.

4: **for** *t* in 1, 2, . . . , *T* **do**

5:    Sever distributes Θ_*t*_ and ${{{{{{{{\mathcal{D}}}}}}}}}_{d}^{t}$ to each client

6:    Server randomly selects a group of clients ${{{{{{{{\mathcal{G}}}}}}}}}_{t}$

7:    **for** each client $c\in {{{{{{{{\mathcal{G}}}}}}}}}_{t}$ (in parallel) **do**

8:       Locally train model Θ_*t*_ on the local dataset ${{{{{{{{\mathcal{D}}}}}}}}}_{l}^{c}$

9:       **for** *i* in 1, 2, . . . , *K* **do**

10:      Compute local model prediction ${{{{{{{{\bf{y}}}}}}}}}_{c,i}^{t}$ on the KT data ${x}_{i}^{t}$

11:        Randomly draw $R \sim {{{{{{{\mathcal{B}}}}}}}}(\beta )$ and ${{{{{{{\bf{f}}}}}}}} \sim {{{{{{{\mathcal{P}}}}}}}}(C)$

12:      Compute perturbed local model prediction ${\hat{{{{{{{{\bf{y}}}}}}}}}}_{c,i}^{t}$ via Eq. (2)

13:       **end** **for**

14:       Upload perturbed local model predictions to the server

15:    **end** **for**

16:    Server aggregates local knowledge and stores them in the knowledge buffer of size *B*

17:    Server fine-tunes the global model Θ_*t*_ on the knowledge buffer

18:    Server self-trains the global model and builds the updated model Θ_*t*+1_

19:   Server samples knowledge transfer data ${{{{{{{{\mathcal{D}}}}}}}}}_{d}^{t+1}$ via the importance sampling mechanism

20: **end** **for**

### Discussion on privacy protection

Next, we will discuss the privacy guarantees of the knowledge transfer in PrivateKT. In PrivateKT, the local private data is kept by each client and never shared with the outside. In order to transfer knowledge from decentralized data to an intelligent model, PrivateKT extracts knowledge from local data into predictions on small KT data, and shares them with a central server for knowledge aggregation. Thus, in PrivateKT, among all local variables correlated to the private data, only local predictions are shared with the server. Since the communication of local predictions may leak raw data, we propose to perturb each local prediction before sending it to the central server to protect user privacy. The privacy security of a single knowledge transfer round in PrivateKT is guaranteed by the *ϵ*-LDP based on Lemma 1. (The proof is in the Supplementary Information.)

#### Lemma 1

Given the size of knowledge transfer samples (i.e., *K*), the privacy protection of knowledge transfer in PrivateKT is gauranteed by *ϵ*-LDP if the following equation holds:

6 $$\beta=\frac{\exp (\frac{\epsilon }{K})-1}{\exp (\frac{\epsilon }{K})-1+C}.$$

Moreover, in PrivateKT we can further avoid the accumulation of privacy budgets during different knowledge transfer rounds based on the model shuffling method^41,57^. Thus, the privacy security of the whole knowledge transfer process in PrivateKT is also guaranteed by *ϵ*-LDP, if the condition in Lemma 1 can be satisfied.

### Reporting summary

Further information on research design is available in the Nature Portfolio Reporting Summary linked to this article.

## Supplementary information

Supplementary Information

Peer review file

Reporting Summary

## Data Availability

The datasets involved in this study are all publicly available ones. The usage of these datasets in this paper is permitted under their licenses. The MNIST dataset is available at http://yann.lecun.com/exdb/mnist/. The MedText dataset is available at https://www.kaggle.com/datasets/chaitanyakck/medical-text. The X-Ray dataset is available at https://www.kaggle.com/datasets/paultimothymooney/chest-xray-pneumonia. The CIFAR-10 and CIFAR-100 datasets are available at https://www.cs.toronto.edu/~kriz/cifar.html. The experimental results generated in this study are provided in the Source Data file. Source data are provided with this paper.

## Source data

Source Data

## Code availability

Codes for this work are available on a public repository^58^ https://github.com/taoqi98/PrivateKT. We also provide sufficient details in the Methods and Supplementary Information for implementing experiments in this work.
