# Supplementary material for: Differentially private knowledge transfer for federated learning
Source: Nat Commun. 2023 Jun 24;14:3785. doi: 10.1038/s41467-023-38794-x (PMC10290720; doi:10.1038/s41467-023-38794-x)
Supplement: Supplementary file 1 — Supplementary Information [file 41467_2023_38794_MOESM1_ESM.pdf]

Supplementary Information for "Differentially Private Knowledge Transfer for Federated Learning"

Tao Qi[1], **Fangzhao Wu**[2*], **Chuhan Wu**[1*], **Liang He**[1], **Yongfeng Huang**[1,3,4*], **and Xing Xie**[2]

[1]Department of Electronic Engineering, Tsinghua University, Beijing 100084, China
[2]Microsoft Research Asia, Beijing 100080, China
[3]Zhongguancun Laboratory, Beijing 100094, China
[4]Institute for Precision Medicine, Tsinghua University, Beijing 102218, China
[*]Correspondence: fangzwu@microsoft.com, wuchuhan15@gmail.com, yfhuang@tsinghua.edu.cn

Supplementary Materials

Dataset Descriptions

The details of experimental datasets are summarized as the follows. These datasets are publicly accessible and the usage of them is permitted under their licenses. The first dataset is MNIST[1], a widely used federated learning benchmark dataset (available at http://yann.lecun.com/exdb/mnist/). The original MNIST dataset includes a training dataset of size 60,000 and a test dataset of size 10,000. The second dataset is a public medical text classification dataset (named MedText). It is released on a Kaggle medical text mining competition, which is available at https://www.kaggle.com/datasets/chaitanyakck/medical-text. The original MedText dataset includes 14,438 medical texts without partition. Thus, we randomly partition 20% of them into the test dataset and the remaining data is used for model training. The third dataset is a public medical image classification dataset. It is released in a Kaggle X-Ray analysis competition available at https://www.kaggle.com/datasets/paultimothymooney/chest-xray-pneumonia. The original X-Ray dataset includes a training dataset of size 5,216 and a test dataset of size 624. Following previous work[2], for each dataset, 20% of training data is used as the candidate unlabeled data pool to choose samples for knowledge transfer, and the remaining data is used for local model training. Moreover, the hyper-parameters of both PrivateKT and baseline methods are selected based on cross-validation.

To simulate the setting of federated learning, the training data of each dataset is randomly partitioned into multiple clients. The data partition is based on two strategies, i.e., independent identical data distribution (IID) and non-identical independent data distribution (Non-IID). For the IID data distribution, each training data is randomly partitioned into each client with an equal probability. The non-IID data partitions are based on three different strategies, including, the class non-IID data partition strategy, size non-IID data partition strategy, and mixed non-IID data partition strategy. Following previous work[3], under the class Non-IID data distribution, the class distributions of local data in different clients are different. The detailed data partition process is summarized in Algorithm 1, where a hyper-parameter α in Algorithm 1 controls the degree of the distribution imbalance and a smaller α means a more imbalanced data distribution. In the size non-IID data distribution, we assume that the local data sizes of different clients are imbalanced and follow the long-tail distribution. Thus, we use the Laplace distribution to randomly generate the local data size. The detailed data partition algorithm is summarized in Algorithm 2, where the hyper-parameter α in Algorithm 2 also controls the degree of the distribution imbalance and a smaller α means a more imbalanced data distribution. For the mixed non-IID data distribution, both the local data class and local data size are imbalanced. The corresponding data partition Algorithm 3 is based on the combination of Algorithm 1 and Algorithm 2. Besides, the detailed statistics of these datasets are summarized in Supplementary Table 1 and 2.

Experimental Settings

Basic Models

In experiments, we employ different knowledge transfer methods (e.g., FedAvg[4] and PrivateKT) to collaboratively train a shared basic model from decentralized data. The basic models for the image classification task in MNIST and X-Ray is based on the convolutional network[1] (named ConvNet). The basic model for the text classification task on MedText is based on the transformer network[5] (named Transformer). Detailed settings are summarized as follows:

ConvNet: The ConvNet model used in the image classification task is the stack of a two-layer 2D-CNN network and an MLP network. The first layer of the 2D-CNN network is set to 64 channels with a 2×2 max-pooling operation. The second layer of the 2D-CNN network is set to 128 channels with a 2×2 max-pooling operation. The kernels of these two layers are set to 5×5. The final MLP network contains four layers, which generate 512-, 512-, 512- and C-dimensional hidden vectors, respectively, where C is the number of classification categories ($C=10$ in MNIST and $C=2$ in X-Ray). The parameters of ConvNet are randomly initialized.

Transformer: The Transformer model used in the text classification task is the stack of a word embedding layer, a transformer network, an attentive pooling network, and an MLP network. The word embedding is initialized by 300-dimensional Glove embeddings[6]. The transformer network is set to contain 20 attention heads, and each head generates 20-dimensional output vectors. The attention network is implemented by a two-layer MLP network with 128-dimensional hidden vectors. The final MLP network contains two layers, and the dimension of output vectors in each layer is 256 and 5, respectively. We use the public glove word embeddings for initializing the word embedding layer, which is available at https://nlp.stanford.edu/data/glove.840B.300d.zip. Besides, other parameters in Transformer are randomly initialized.

Hyperparameter Settings

Next, we will introduce the hyper-parameter settings of PrivateKT in experiments. In each round of federated learning, we randomly select 50% clients for knowledge transfer. We use the SGD optimizer[1] with 0.05 learning rate for local model training on MNIST and X-Ray. We use the Adam optimizer[7] with 0.0001 learning rate for local model training on MedText. The

size of knowledge transfer samples is set to 2 and the privacy budget ε is set to 5, which can further determine the random response probability β based on Lemma 3. Besides, the size of the knowledge buffer is set to 25. We sample 10% data from the unlabeled dataset in the server for self-training. For each dataset, the optimization strategies for the model updating on the server are the same as the optimization strategies for the local model training. In addition, in order to reduce the model overfitting, we apply the dropout method[8] with 0.2 dropout probability to the output of each layer in the basic models. The complete hyper-parameter settings are summarized in Supplementary Table 3.

Metrics

Since the classification task in MNIST is balanced, we use accuracy as the evaluation metric. Besides, since the classification tasks in MedText and X-Ray are imbalanced, we use the Macro-F1 for performance verification. To compute Macro-F1, we first compute the F1-score for each classification category and then average them as the Macro-F1 score. The computations of these metrics are summarized as follows:

$$\mathrm{Accuracy} = \frac{1}{|\mathscr{D}_\mathrm{e}|}\sum_{\mathrm{i}=1}^{|\mathscr{D}_\mathrm{e}|} \mathrm{I}[\mathrm{y_i} = \hat{\mathrm{y}}_\mathrm{i}], \tag{1}$$

$$\mathrm{Precision_i} = \frac{\mathrm{TP_i}}{\mathrm{TP_i} + \mathrm{FP_i}}, \tag{2}$$

$$\mathrm{Recall_i} = \frac{\mathrm{TP_i}}{\mathrm{TP_i} + \mathrm{FN_i}}, \tag{3}$$

$$\mathrm{F1_i} = \frac{2 * \mathrm{Precision_i} * \mathrm{Recall_i}}{\mathrm{Precision_i} + \mathrm{Recall_i}}, \tag{4}$$

$$\mathrm{Macro-F1} = \frac{1}{\mathrm{C}}\sum_{\mathrm{i}=1}^{\mathrm{C}} \mathrm{F1_i}, \tag{5}$$

where $\mathscr{D}_e$ is the test dataset, y_i and $\hat{y}_i$ is the ground truth and the model prediction of the i-th test data in $\mathscr{D}_e$. $\mathrm{I}[\cdot]$ is the indicator function. $\mathrm{TP_i}$, $\mathrm{FP_i}$ and $\mathrm{FN_i}$ are true positive, false positive, and false negative rate, of the test data belonging to the i-th category, respectively. $\mathrm{Precision_i}$, $\mathrm{Recall_i}$, and $\mathrm{F1_i}$ denote the Precision, Recall, and F1 score of the test data belonging to the i-th category, respectively. C denotes the number of classification categories.

Comparisons on Efficiency

In this section, we compare the efficiency of different methods. We first compare the communication efficiency of PrivateKT and existing FL methods. We use the size of data exchanged between a client and the server in a round to measure the communication costs, and present the upload and the download communication costs of different methods (Fig. 1). First, we find that PrivateKT can maximally reduce 99% of the upload communication costs. This is because existing FL methods usually transfer the knowledge from local data to the global model, by uploading a large volume of variables (such as local model updates) to the server. By contrast, PrivateKT transfers knowledge via small actively selected public data, and thereby can effectively reduce the upload communication costs. Second, we find that PrivateKT is comparable with existing FL methods in download communication costs. This is because, in each round of federated learning, local clients need to download the latest global model from the server, which is the bottleneck of the download communication costs in different methods. Although PrivateKT needs to extra communicate some knowledge transfer data in each round, the extra cost is usually negligible compared with the cost caused by communicating model parameters. This is because in a single round PrivateKT only requires clients to download a very small amount of knowledge transfer data (e.g., 2) from the server, and the size of a single data is usually small (e.g., 5kB). For example, in our experiments, the cost of communicating knowledge transfer data is less than 10kB, which is much smaller than the cost of communicating parameters (e.g., 6.4 MB). In our future work, we plan to improve PrivateKT by communicating the aggregated knowledge from the server to clients, rather than the updated model parameters, and locally fine-tuning the intelligent model, to further reduce the download communication costs.

Next, we compare the computing efficiency of different methods on the local clients and the central server. For local clients, baseline FL methods require them to locally train the intelligent model. The corresponding computational complexity is $\mathscr{O}(Nd^3)$, where d is the number of model parameters and N is the number of local training samples. Besides local model

training, PrivateKT extra requires the local clients to infer the pseudo label for the knowledge transfer data. The corresponding computational complexity is $\mathcal{O}(nd^2)$, where n is the number of knowledge transfer samples. Since in applications n (e.g., 2) is usually much smaller than N (e.g., 100), the computing cost of PrivateKT is still dominated by the local model training, and its extra computation cost is usually negligible. For the central server, baseline FL methods usually require it to aggregate the local model parameters. The corresponding computational complexity is $\mathcal{O}(Hd)$, where H denotes the number of participant clients in this round. By contrast, the bottleneck computing cost of PrivateKT is training the global model on the knowledge buffer and the self-training samples. The computational complexity of PrivateKT on the server is $\mathcal{O}((M+B)d^3)$, where M denotes the number of the self-training samples and B denotes the knowledge buffer size. Thus, the computing cost of PrivateKT for the server is more expensive than that of baseline methods. Fortunately, in real-world scenarios, the central server usually contains rich computing resources and the extra computing cost is usually acceptable. The detailed computational complexities of different methods are summarized in Table 4.

Finally, we compare the storage costs of different methods. Compared with baseline FL methods, PrivateKT requires more storage in the server to maintain the knowledge buffer. The extra storage cost is proportional to the size of the unlabeled knowledge dataset. Fortunately, in real-world practice, the size of the knowledge transfer dataset is usually moderate. For example, in our experiments, the extra storage cost of PrivateKT is 8.97 MB, 0.88 MB, and 2.59 MB on the MNIST, MedText, and X-Ray datasets, respectively. Thus, the extra storage cost of PrivateKT is usually acceptable for a modern central server.

Comparisons on Generality

Next, we compare the generality and scalability of different methods. In our previous experiments, we only trained a single backbone model on each dataset to compare different methods. It may arouse concerns that the conclusions based on a single backbone model may be not generalized. Thus, we further train other backbone models for each dataset to verify the generality of PrivateKT. The backbone model for the image dataset is ResNet-50[9] and the backbone model for the text dataset is BERT[10]. These two backbone models contain much more parameters than our previous backbone models, which can be also used to verify the scalability of PrivateKT. We compare different methods under the LDP privacy guarantees and the class Non-IID data distribution (Fig. 2). First, we find that the performance of baseline FL methods still degrades into random guesses. This is because the backbone models contain more parameters and these methods need stronger LDP noise to protect privacy. Second, we find that PrivateKT can effectively train big models with effective privacy protection. This is because PrivateKT utilizes small data to extract and transfer knowledge from big models. It is still effective for the LDP technique to protect the knowledge carried by small data. These results verify the generality and scalability of PrivateKT.

Comparisons with Baseline Variants

Next, we compare PrivateKT with the variants of baseline FL methods for further evaluation. An unlabeled public dataset is used in PrivateKT for knowledge transfer, while it is not used in many traditional FL methods (e.g., FedSGD and FedAdam), which may bring an unfair advantage to PrivateKT. Thus, for fairer comparisons, we implement the variants of baseline methods that fine-tune the aggregated global model on the unlabeled dataset via self-training, and compare them with PrivateKT. The experiments are based on the protection of LDP and class non-IID data distribution (Fig. 3). We find that the unlabeled data and the self-training technique can hardly improve the performance of baseline FL methods. This is because the LDP noise usually seriously hurts the accuracy of the aggregated model, making it difficult for the aggregated model to generate accurate pseudo-labels for public data. Furthermore, the self-training based on inaccurate pseudo-labels is usually ineffective and can hardly enhance the model performance. These results further show the superiority of PrivateKT over baseline methods.

Impact of the Knowledge Transfer Data Distribution

Next, we compare the impact of the knowledge transfer data distribution on PrivateKT. The distribution difference between the knowledge transfer data and the local training data is set to three-level, i.e., IID, weak non-IID, and strong non-IID. In the IID setting, we randomly partition 20% of the training data of the original training dataset as the public dataset and the remaining training data is used for local model training. The non-IID data partition strategy follows Algorithm 1, where α is set to 10 and 1 for the weak and strong non-IID settings respectively. From the results in Fig. 4, we find that an unlabeled public dataset whose distribution is similar to the training data can benefit the knowledge transfer in PrivateKT. Besides, we also find that the performance drop of PrivateKT is minor when there is gap between the distributions of public data and training data. These results indicate that PrivateKT can still achieve good performance in real-world scenarios where the distributions of the public data and the local training data are different.

Impact of the Candidate Public Data Pool Size

Since in some scenarios it may be not easy to obtain a large unlabeled dataset that can be shared across different parities for knowledge transfer, which may limit the application of PrivateKT. Thus, we further explore how the size of the candidate public data pool affects model performance under various privacy guarantees (Fig. 5). Results show that collecting more public

data improves the performance of PrivateKT under the law of diminishing returns[11] . For example, on the MedText dataset, the Macro-F1 of PrivateKT can increase about 9% when the public data size increases from 100 to 500, while the Macro-F1 can only increase about 1% when the public data size increases from 1000 to 2000, Moreover, we also find that PrivateKT is still effective when the knowledge transfer data can be actively sampled from only a small amount of public data. For example, the accuracy of PrivateKT is around 90% on MNIST when the size of the public data pool is only 100. These results show PrivateKT is robust to the amount of candidate public data and show its potential in scenarios with only a small amount of public data. Moreover, some existing works show that knowledge transfer can be performed in a data-free manner[12–14]. Thus, in our future work, we plan to apply these methods to PrivateKT to improve its accessibility in real-world scenarios.

Impact of the Knowledge Buffer Size

Next, we analyze the impact of the knowledge buffer size on the effectiveness of knowledge transfer (Fig. 6). First, we find that with the increase of the buffer size, the performance of PrivateKT is effectively improved at the beginning. For example, the Macro-F1 of PrivateKT increases around 10% on MedText when the buffer size increases from 1 to 25. This is because in PrivateKT, to mitigate the damage of LDP noise on model performance, only a small amount of data can be used for knowledge transfer in each round. However, small data may be insufficient for effective knowledge transfer. To tackle this challenge, we propose a knowledge buffer that stores aggregated knowledge in the previous several rounds for updating the global model. By increasing the knowledge buffer size, more useful knowledge can be encoded into the global model to enhance knowledge transfer. Second, we find that the effectiveness of PrivateKT is also suboptimal when the knowledge buffer size is too large. For example, the Macro-F1 of PrivateKT declines around 10% on MedText when the buffer size increases from 25 to 150. This is because a knowledge buffer in large size stores aggregated knowledge from many rounds ago, which may be inaccurate for the training of the latest global model. Thus, a moderate knowledge buffer size, e.g. 25 and 50, is more suitable for PrivateKT.

Impact of the Self-Training Sample Size

Next, we analyze the impact of the self-training sample size (i.e., the hyper-parameter M) on the performance of PrivateKT (Fig. 7). We find that with the increase of M the performance of PrivateKT nearly remains unchanged, which shows that M is not a sensitive hyper-parameter for PrivateKT.

Bias Analysis of PrivateKT

Next, we analyze the potential bias of PrivateKT. In PrivateKT, we only select a small amount of data from the public dataset for knowledge transfer. The data selection in PrivateKT may have the risk of introducing bias to the model training when the distribution of local data is non-IID. Thus, in order to verify the unbiasedness of PrivateKT, we compare the bias of the model learned by PrivateKT and two baseline FL methods that transfer knowledge based on full public data (i.e., FedED and FedMD). We evaluate the model bias by computing its performance deviations across test data in different clients (Fig. 8). We find that there is no significant difference between the performance deviation of PrivateKT and other methods. This is because, although in a single training round the knowledge carried by small data may be biased for the model construction, federated learning is iterated for multiple rounds. Even though some data is not fully explored for knowledge transfer at the beginning of model learning, PrivateKT can also provide more opportunities for these samples in the subsequent model training based on the proposed importance sampling mechanism. Thus, compared with baseline FL methods, the small data-based knowledge transfer in PrivateKT does not lead to additional bias in the model training. In addition, based on Fig. 8, we note that different methods usually have a little performance deviation when local data is non-IID. This is because some classification categories are harder than other categories for models to classify. When the local data is non-IID, some clients may store more hard samples, which may decrease the model performance for them. By contrast, some clients may store fewer hard samples, which makes the model have better performance for them. Finally, performance deviation of the model on different clients is increased.

Evaluation on Larger Datasets

Next, we further evaluate the effectiveness of PrivateKT and baseline methods on larger FL benchmark datasets, i.e., CIFAR-10 and CIFAR-100 (Fig. 9). The comparisons are based on Non-IID settings. From the results, we first find that, compared with the privacy-invasive centralized training, PrivateKT faces more significant performance degradation on larger datasets. This is because, according to the no free lunch theorem for privacy security and algorithm utility of federated learning[15], stronger privacy protection will lead to poorer algorithm performance. Training FL models on a larger volume of data usually needs more effort on privacy protection, and results in more serious performance degradation. Second, we find that PrivateKT still effectively improves the performance of baseline FL methods under serious privacy restrictions. This phenomenon demonstrates the main conclusions of our work still hold on larger datasets. It also further demonstrates that PrivateKT is a more promising privacy-preserving machine learning framework than other methods in most real-world scenarios with small and moderately large volumes of training data.

Discussions on the Robustness of PrivateKT

In real-world applications, existing federated learning systems are usually exposed to an untrustworthy environment, which may be attacked by some malicious parties. The malicious attack can threaten the security of our PrivateKT method and limit its application in real-world scenarios. Thus, we will briefly discuss the robustness issue of PrivateKT and the potential robustness mechanism for it. First, some existing works reveal that the LDP noise can improve model robustness to some extent[16]. In PrivateKT, we utilize the LDP technique to perturb the exchanged knowledge, which may improve the robustness of our method. Second, compared with attacking big data-based knowledge transfer methods, the feasible malicious modification operations on the small data are usually much less. Moreover, to achieve a comparable attack success rate, poisoning small data is usually less covert than poisoning big data. These two facts indicate that it may be more difficult to attack a small data-based knowledge transfer method than other big data-based knowledge transfer methods. In addition, according to the second fact, robust knowledge aggregation methods[17,18] by filtering the outlier local knowledge is suitable for protecting PrivateKT from attack. Since the model robustness problem is out of the scope of this work, we plan to design a robust knowledge aggregation method for PrivateKT in our future work.

Discussions on the Utility and Privacy of PriavteKT

Next, we present some detailed analysis on the knowledge utility and privacy guarantees of PrivateKT. In order to encode the local knowledge of different clients into the global model, PrivateKT updates the global model based on the estimated averaged predictions of different clients on the knowledge transfer data. Recall that the estimation $\hat{\mathbf{y}}_i^t$ of the averaged prediction $\mathbf{y}_i^t = \frac{1}{|\mathcal{G}_t|}\sum_{c\in\mathcal{G}_t}\mathbf{y}_{c,i}^t$ for the knowledge transfer data x_i^t is based on the following equation:

$$\hat{\mathbf{y}}_i^t = \frac{1}{\beta}\left(\frac{1}{|\mathcal{G}_t|}\sum_{c\in\mathcal{G}_t}\hat{\mathbf{y}}_{c,i}^t - \frac{1-\beta}{C}\mathbf{1}\right), \tag{6}$$

where $\mathcal{G}_t$ is the set of clients that participate in the t-th knowledge transfer round, $\mathbf{y}_{c,i}^t$ is the local model prediction of the client c on x_i^t, and $\hat{\mathbf{y}}_{c,i}^t$ is the perturbed local prediction of the client c on x_i^t. Since the exchanged local model predictions are perturbed to protect user privacy, the accuracy of the knowledge estimation can substantially impact the knowledge utility of PrivateKT. Lemma 1 and Lemma 2 show that $\hat{\mathbf{y}}_i^t$ is an unbiased estimation of $\mathbf{y}_i^t$ and the mean square error of the estimation can asymptotically converge to 0, which guarantee that the damage of LDP noise on model performance can be effectively mitigated in PrivateKT.

Supplementary Lemma 1: $\hat{\mathbf{y}}_i^t$ is an unbiased estimation of $\mathbf{y}_i^t$: $\mathbb{E}[\,\hat{\mathbf{y}}_i^t\,] = \mathbf{y}_i^t$.

Supplementary Lemma 2: The mean square error of $\hat{\mathbf{y}}_i^t$ is bounded by $\frac{(C-1)(1-\beta^2)}{|\mathcal{G}_t|C\beta^2}$ and can asymptotically converge to 0:

$$\mathbb{E}[\,||\hat{\mathbf{y}}_i^t - \mathbf{y}_i^t||_2^2\,] < \frac{(C-1)(1-\beta^2)}{|\mathcal{G}_t|C\beta^2}, \tag{7}$$

$$\lim_{|\mathcal{G}_t|\to\infty}\mathbb{E}[\,||\hat{\mathbf{y}}_i^t - \mathbf{y}_i^t||_2^2\,] = 0. \tag{8}$$

Besides, as discussed in our main paper, the privacy protection of PrivateKT can be guaranteed by ε-LDP based on Lemma 3.

Supplementary Lemma 3: Given the size of knowledge transfer samples (i.e., K), the privacy protection of knowledge transfer in PrivateKT is gauranteed by ε-LDP if the following equation holds:

$$\beta = \frac{\exp(\frac{\varepsilon}{K}) - 1}{\exp(\frac{\varepsilon}{K}) - 1 + C}. \tag{9}$$

Besides, according to Lemma 2 and Lemma 3, we find that the knowledge utility and privacy guarantee of PrivateKT is connected by the random response probability β. This result can provide theoretical guidance on how to balance the privacy protection and knowledge utility in PrivateKT. Next, we will give the proofs of these Lemmas.

Supplementary Proof 1 (Proof of Supplementary Lemma 1): First, the expectation of $\hat{\mathbf{y}}_i^t$ can be formulated as follow:

$$\mathbb{E}[\,\hat{\mathbf{y}}_i^t\,] = \mathbb{E}[\,\frac{1}{\beta}(\frac{1}{|\mathcal{G}_t|}\sum_{c\in\mathcal{G}_t}\hat{\mathbf{y}}_{c,i}^t - \frac{1-\beta}{C}\mathbf{1})] = \frac{1}{\beta}(\frac{1}{|\mathcal{G}_t|}\sum_{c\in\mathcal{G}_t}\mathbb{E}[\,\hat{\mathbf{y}}_{c,i}^t\,] - \frac{1-\beta}{C}\mathbf{1}). \tag{10}$$

Recall that $\hat{\mathbf{y}}^t_{c,i}$ can be represented as the combination of true local prediction $\mathbf{y}^t_{c,i}$ and a randomly-generated category label $\mathbf{f}$:

$$\hat{\mathbf{y}}^t_{c,i} = R \cdot \mathbf{y}^t_{c,i} + (1-R) \cdot \mathbf{f}, \tag{11}$$

where $\mathbf{y}^t_{c,i} \in \{0,1\}^C$, $\mathbf{f} \in \{0,1\}^C \sim \mathscr{P}(C)$, $R \sim \mathscr{B}(\beta)$, C is the number of classification categories and β is the probability of assigning R to 1. Based on the linearity of expectation, the following equation holds:

$$\mathbb{E}[\,\hat{\mathbf{y}}^t_{c,i}\,] = \mathbb{E}[\,R \cdot \mathbf{y}^t_{c,i} + (1-R) \cdot \mathbf{f}\,] = \mathbb{E}[\,R\,] \cdot \mathbf{y}^t_{c,i} + (1-\mathbb{E}[\,R\,]) \cdot \mathbb{E}[\,\mathbf{f}\,] = \beta \cdot \mathbf{y}^t_{c,i} + \frac{1-\beta}{C}\mathbf{1}. \tag{12}$$

Thus, based on Equation 10 and Equation 12, we can prove Lemma 1:

$$\mathbb{E}[\,\hat{\mathbf{y}}^t_i\,] = \frac{1}{\beta}\Big(\frac{1}{|\mathscr{G}_t|}\sum_{c\in\mathscr{G}_t}\mathbb{E}[\,\hat{\mathbf{y}}^t_{c,i}\,] - \frac{1-\beta}{C}\mathbf{1}\Big) = \frac{1}{|\mathscr{G}_t|}\sum_{c\in\mathscr{G}_t}\mathbf{y}^t_{c,i} = \mathbf{y}^t_i. \tag{13}$$

Supplementary Proof 2 (Proof of Supplementary Lemma 2): For an arbitrary KD data x^t_i, the mean square error $\mathbb{E}[\,||\hat{\mathbf{y}}^t_i - \mathbf{y}^t_i||^2_2\,]$ of the estimation $\hat{\mathbf{y}}^t_i$ on $\mathbf{y}^t_i$ can be formulated as the follow:

$$\begin{aligned}
\mathbb{E}[\,||\hat{\mathbf{y}}^t_i - \mathbf{y}^t_i||^2_2\,] &= \mathbb{E}[\,||\frac{1}{\beta}\Big(\frac{1}{|\mathscr{G}_t|}\sum_{c\in\mathscr{G}_t}\hat{\mathbf{y}}^t_{c,i} - \frac{1-\beta}{C}\mathbf{1}\Big) - \frac{1}{|\mathscr{G}_t|}\sum_{c\in\mathscr{G}_t}\mathbf{y}^t_{c,i}||^2_2\,] \\
&= \mathbb{E}[\,||\frac{1}{|\mathscr{G}_t|}\sum_{c\in\mathscr{G}_t}\Big(\frac{1}{\beta}\hat{\mathbf{y}}^t_{c,i} - \frac{1-\beta}{\beta C}\mathbf{1}\Big) - \frac{1}{|\mathscr{G}_t|}\sum_{c\in\mathscr{G}_t}\mathbf{y}^t_{c,i}||^2_2\,] \\
&= \mathbb{E}[\,||\frac{1}{|\mathscr{G}_t|}\sum_{c\in\mathscr{G}_t}\Big(\frac{1}{\beta}\hat{\mathbf{y}}^t_{c,i} - \frac{1-\beta}{\beta C}\mathbf{1} - \mathbf{y}^t_{c,i}\Big)||^2_2\,] \\
&= \frac{1}{|\mathscr{G}_t|^2\beta^2}\mathbb{E}[\,||\sum_{c\in\mathscr{G}_t}\Big(\hat{\mathbf{y}}^t_{c,i} - \frac{1-\beta}{C}\mathbf{1} - \beta\mathbf{y}^t_{c,i}\Big)||^2_2\,].
\end{aligned} \tag{14}$$

Note that L2 norm $||\cdot||^2_2$ is a convex function, then the following inequation always holds:

$$\begin{aligned}
\mathbb{E}[\,||\hat{\mathbf{y}}^t_i - \mathbf{y}^t_i||^2_2\,] &= \frac{1}{|\mathscr{G}_t|^2\beta^2}\mathbb{E}[\,||\sum_{c\in\mathscr{G}_t}\Big(\hat{\mathbf{y}}^t_{c,i} - \frac{1-\beta}{C}\mathbf{1} - \beta\mathbf{y}^t_{c,i}\Big)||^2_2\,] \\
&< \frac{1}{|\mathscr{G}_t|^2\beta^2}\sum_{c\in\mathscr{G}_t}\mathbb{E}[\,||\hat{\mathbf{y}}^t_{c,i} - \frac{1-\beta}{C}\mathbf{1} - \beta\mathbf{y}^t_{c,i}||^2_2\,] \\
&= \frac{1}{|\mathscr{G}_t|^2\beta^2}\sum_{c\in\mathscr{G}_t}\{\mathbb{E}[\,||\hat{\mathbf{y}}^t_{c,i}||^2_2\,] + \mathbb{E}[\,||\frac{1-\beta}{C}\mathbf{1} + \beta\mathbf{y}^t_{c,i}||^2_2\,] - 2\mathbb{E}[\,\hat{\mathbf{y}}^t_{c,i}\,]^T\mathbb{E}[\,\frac{1-\beta}{C}\mathbf{1} + \beta\mathbf{y}^t_{c,i}\,]\} \\
&= \frac{1}{|\mathscr{G}_t|^2\beta^2}\sum_{c\in\mathscr{G}_t}\{\,\mathbb{E}[\,||\hat{\mathbf{y}}^t_{c,i}||^2_2\,] - ||\frac{1-\beta}{C}\mathbf{1} + \beta\mathbf{y}^t_{c,i}||^2_2\,\}.
\end{aligned} \tag{15}$$

Note that, for an arbitrary $c \in \mathscr{G}_t$ and an arbitrary KT sample x^t_i, the following equitation holds:

$$\begin{aligned}
\mathbb{E}[\,||\hat{\mathbf{y}}^t_{c,i}||^2_2\,] &= \mathbb{E}[\,||\hat{\mathbf{y}}^t_{c,i}||^2_2 \mid R=1\,] \cdot Pr[R=1] + \mathbb{E}[\,||\hat{\mathbf{y}}^t_{c,i}||^2_2 \mid R=0\,] \cdot Pr[R=0] \\
&= \mathbb{E}[\,||\mathbf{y}^t_{c,i}||^2_2 \mid R=1\,] \cdot \beta + \mathbb{E}[\,||\mathbf{f}||^2_2 \mid R=0\,] \cdot (1-\beta) \\
&= 1 \cdot \beta + 1 \cdot (1-\beta) \\
&= 1.
\end{aligned} \tag{16}$$

Besides, for an arbitrary $c \in \mathscr{G}_t$ and an arbitrary KT sample x^t_i, the following equitation holds:

$$\begin{aligned}
||\frac{1-\beta}{C}\mathbf{1} + \beta\mathbf{y}^t_{c,i}||^2_2 &= \frac{(1-\beta)^2}{C^2}||\mathbf{1}||^2_2 + \beta^2||\mathbf{y}^t_{c,i}||^2_2 + \frac{2\beta(1-\beta)}{C}\mathbf{1}^T\mathbf{y}^t_{c,i} \\
&= \frac{(1-\beta)^2}{C^2} \cdot C + \beta^2 \cdot 1 + \frac{2\beta(1-\beta)}{C} \cdot 1 \\
&= \frac{(C-1)\beta^2 + 1}{C}.
\end{aligned} \tag{17}$$

Based on Equation 15, 16, and 17, we can build an upper bound for the estimation error:

$$\begin{aligned}\mathbb{E}[\ ||\hat{\mathbf{y}}_i^t - \mathbf{y}_i^t||_2^2\ ] &< \frac{1}{|\mathcal{G}_t|^2\beta^2}\sum_{c\in\mathcal{G}_t}\{\ \mathbb{E}[\ ||\hat{\mathbf{y}}_{c,i}^t||_2^2\ ] - ||\frac{1-\beta}{C}\mathbf{1} + \beta\mathbf{y}_{c,i}^t||_2^2\ \}\\ &= \frac{1}{|\mathcal{G}_t|^2\beta^2}\sum_{c\in\mathcal{G}_t}\frac{(C-1)(1-\beta^2)}{C}\\ &= \frac{(C-1)(1-\beta^2)}{|\mathcal{G}_t|C\beta^2}.\end{aligned} \tag{18}$$

Note that the following inequation and equation always hold:

$$0 < \mathbb{E}[\ ||\hat{\mathbf{y}}_i^t - \mathbf{y}_i^t||_2^2\ ] < \frac{(C-1)(1-\beta^2)}{|\mathcal{G}_t|C\beta^2}, \tag{19}$$

$$\lim_{|\mathcal{G}_t|\to\infty}\frac{(C-1)(1-\beta^2)}{|\mathcal{G}_t|C\beta^2} = 0, \tag{20}$$

thereby, according to the Squeeze theorem, the mean square error of the estimation can asymptotically converge to 0:

$$\lim_{|\mathcal{G}_t|\to\infty}\mathbb{E}[\ ||\hat{\mathbf{y}}_i^t - \mathbf{y}_i^t||_2^2\ ] = 0. \tag{21}$$

Supplementary Proof 3 (Proof of Supplementary Lemma 3): For two arbitrary local prediction $\mathbf{y}$ and $\mathbf{y}'$, and any legal perturbed local prediction $\hat{\mathbf{y}}$, the following equation always holds:

$$\max\frac{Pr[\mathcal{M}(\mathbf{y}) = \hat{\mathbf{y}}]}{Pr[\mathcal{M}(\mathbf{y}') = \hat{\mathbf{y}}]} = \frac{Pr[\mathcal{M}(\mathbf{y}) = \hat{\mathbf{y}}]|\mathbf{y} = \hat{\mathbf{y}}]}{Pr[\mathcal{M}(\mathbf{y}') = \hat{\mathbf{y}}|\mathbf{y}' \neq \hat{\mathbf{y}}]} = \frac{\beta + (1-\beta)\frac{1}{C}}{(1-\beta)\frac{1}{C}} = \frac{(C-1)\beta+1}{1-\beta}. \tag{22}$$

According to the definition of the local differential privacy, the privacy protection on a single local prediction can meet ε_0-LDP if the following condition holds:

$$\exp(\varepsilon_0) = \max\frac{Pr[\mathcal{M}(\mathbf{y}) = \hat{\mathbf{y}}]}{Pr[\mathcal{M}(\mathbf{y}') = \hat{\mathbf{y}}]} = \frac{(C-1)\beta+1}{1-\beta}. \tag{23}$$

Besides, since each client uploads K perturbed local predictions in each round, the privacy budget for protecting the whole communication should be accumulated. Thus, the privacy protection on the whole data communication is guaranteed by ε-LDP if the privacy protection on a single local prediction is guaranteed by $\frac{\varepsilon}{K}$-LDP, which needs:

$$\exp(\frac{\varepsilon}{K}) = \frac{(C-1)\beta+1}{1-\beta}. \tag{24}$$

Thus, based on Equation 24 the knowledge transfer of PrivateKT can meet ε-LDP if the following condition holds:

$$\beta = \frac{\exp(\frac{\varepsilon}{K}) - 1}{\exp(\frac{\varepsilon}{K}) - 1 + C}. \tag{25}$$

Supplementary References

1. LeCun, Y., Bottou, L., Bengio, Y. & Haffner, P. Gradient-based learning applied to document recognition. *Proc. IEEE* 2278–2324 (1998).

2. Sui, D. *et al.* Feded: Federated learning via ensemble distillation for medical relation extraction. In *EMNLP*, 2118–2128 (2020).

3. Hsu, T.-M. H., Qi, H. & Brown, M. Measuring the effects of non-identical data distribution for federated visual classification. *arXiv preprint arXiv:1909.06335* (2019).

4. McMahan, B., Moore, E., Ramage, D., Hampson, S. & y Arcas, B. A. Communication-efficient learning of deep networks from decentralized data. In *AISTATS*, 1273–1282 (2017).

5. Vaswani, A. *et al.* Attention is all you need. In *NeurIPS*, 5998–6008 (2017).

6. Pennington, J., Socher, R. & Manning, C. Glove: Global vectors for word representation. In *EMNLP*, 1532–1543 (2014).

7. Kingma, D. P. & Ba, J. Adam: A method for stochastic optimization. In *ICLR* (2015).

8. Srivastava, N., Hinton, G., Krizhevsky, A., Sutskever, I. & Salakhutdinov, R. Dropout: A simple way to prevent neural networks from overfitting. *JMLR* 1929–1958 (2014).

9. He, K., Zhang, X., Ren, S. & Sun, J. Deep residual learning for image recognition. In *CVPR*, 770–778 (2016).

10. Kenton, J. D. M.-W. C. & Toutanova, L. K. Bert: Pre-training of deep bidirectional transformers for language understanding. In *NAACL*, 4171–4186 (2019).

11. Shephard, R. W. & Färe, R. The law of diminishing returns. In *Production theory*, 287–318 (Springer, 1974).

12. Nayak, G. K., Mopuri, K. R., Shaj, V., Radhakrishnan, V. B. & Chakraborty, A. Zero-shot knowledge distillation in deep networks. In *ICML*, 4743–4751 (2019).

13. Zhu, Z., Hong, J. & Zhou, J. Data-free knowledge distillation for heterogeneous federated learning. In *ICML*, 12878–12889 (2021).

14. Chawla, A., Yin, H., Molchanov, P. & Alvarez, J. Data-free knowledge distillation for object detection. In *WACV*, 3289–3298 (2021).

15. Zhang, X., Gu, H., Fan, L., Chen, K. & Yang, Q. No free lunch theorem for security and utility in federated learning. *ACM Transactions on Intell. Syst. Technol.* **14**, 1–35 (2022).

16. Sun, Z., Kairouz, P., Suresh, A. T. & McMahan, H. B. Can you really backdoor federated learning? *arXiv preprint arXiv:1911.07963* (2019).

17. Awan, S., Luo, B. & Li, F. Contra: Defending against poisoning attacks in federated learning. In *ESORICS*, 455–475 (2021).

18. Blanchard, P., El Mhamdi, E. M., Guerraoui, R. & Stainer, J. Machine learning with adversaries: Byzantine tolerant gradient descent. vol. 30 (2017).

255 ## Supplementary Figures

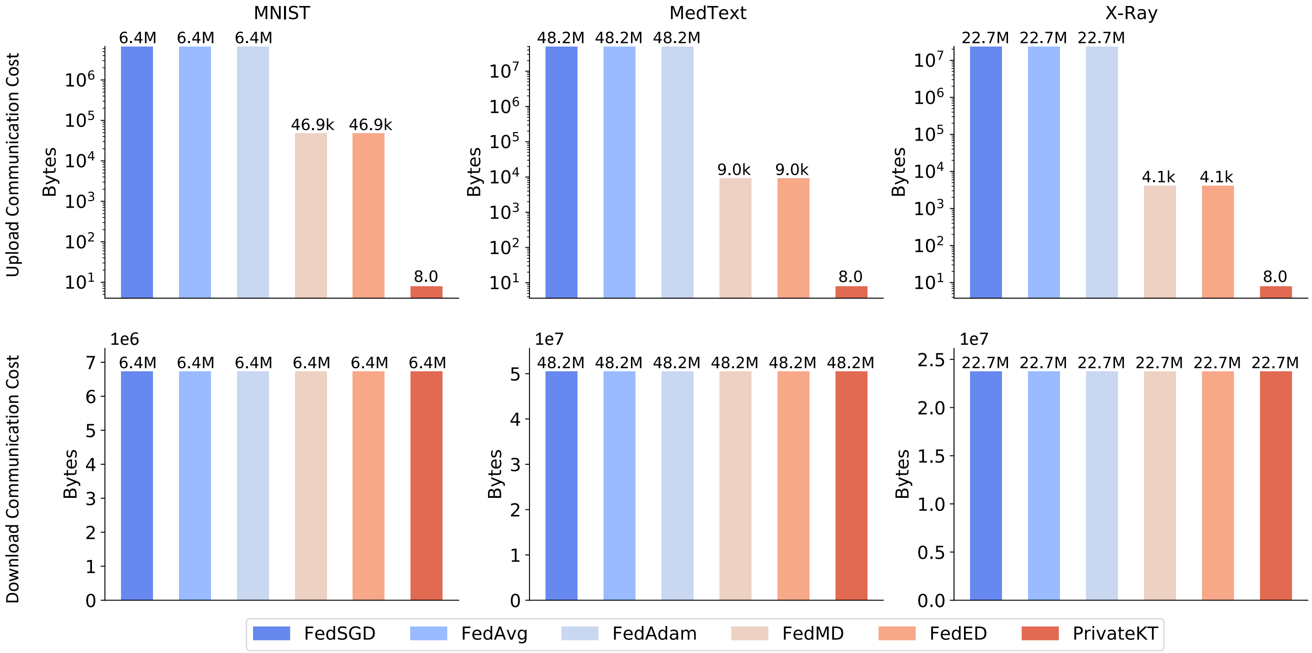

Supplementary Figure 1. Communication efficiency comparisons of different methods. In the figures, we present the upload and download communications costs of a client in a knowledge transfer round, which are measured by the volume of communicated data. (The y-axis for presenting upload communication cost is in logarithmic scale). The results show that PrivateKT can effectively reduce the upload communication costs of existing federated learning methods.

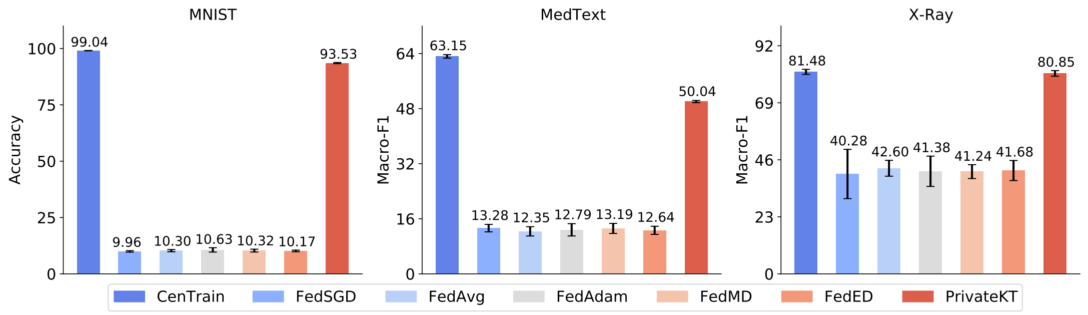

Supplementary Figure 2. Generality comparisons of different methods. The error bars represent the mean results with standard deviations. We evaluate the performance of different methods for learning big models. Results show that PrivateKT can effectively train machine learning models with massive parameters under strict privacy guarantees.

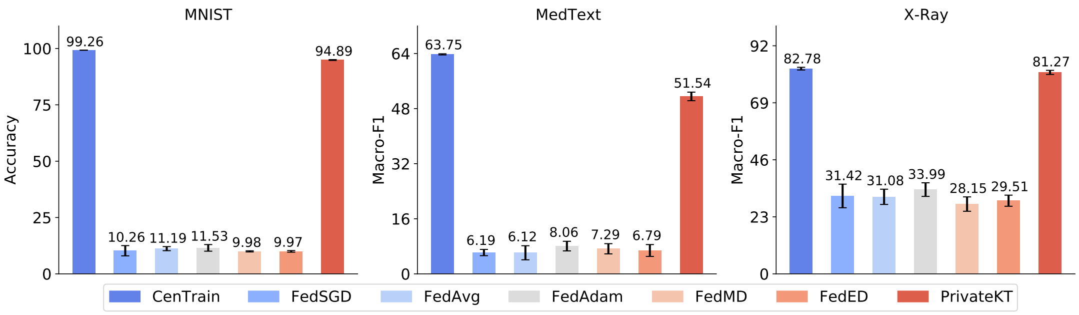

Supplementary Figure 3. Comparisons between PrivateKT and variants of baseline FL methods. The error bars represent the mean results with standard deviations. For fairer comparisons, we implement the variations of baseline FL methods that use the unlabeled public dataset for self-training and compare them with PrivateKT. Results show that baseline methods still degrade into random guesses and PrivateKT significantly outperforms them.

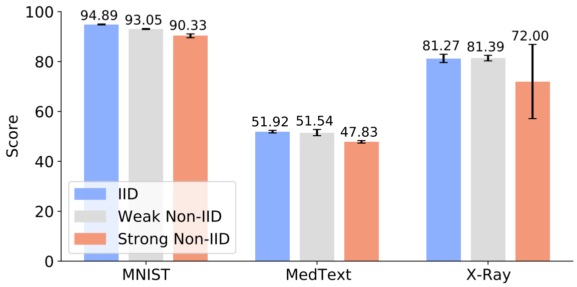

Supplementary Figure 4. The influence of the knowledge transfer data distribution. The error bars represent the mean results with standard deviations. We evaluate the performance of PrivateKT under different knowledge transfer data distributions. The results show that PrivateKT is more effective when the knowledge transfer data distribution is more similar to the training data.

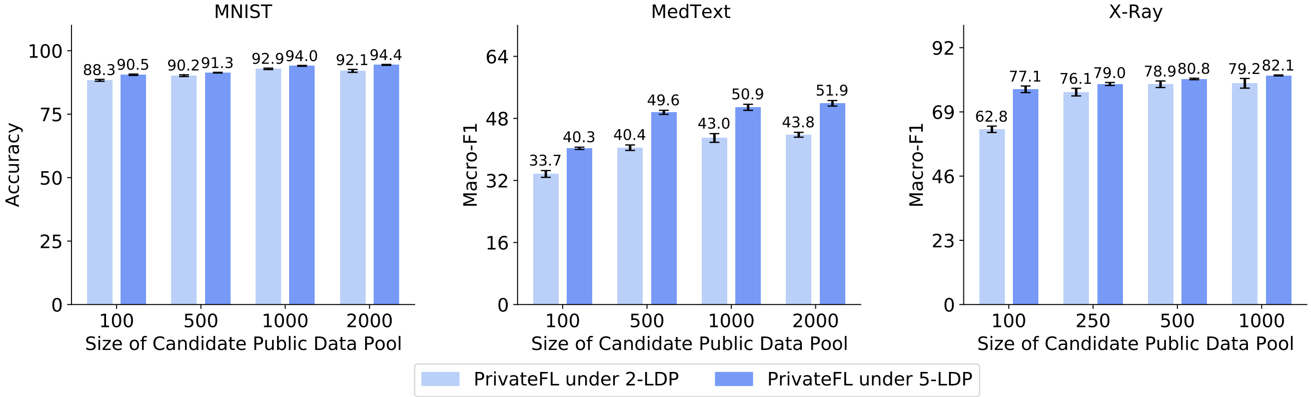

Supplementary Figure 5. The performance of PrivateKT under different amounts of public data. The error bars represent the mean results with standard deviations. The results show that PrivateKT can still achieve effective performance when only scarce (e.g., 100) public data can be used for knowledge transfer, indicating the robustness of PrivateKT on the amount of public data.

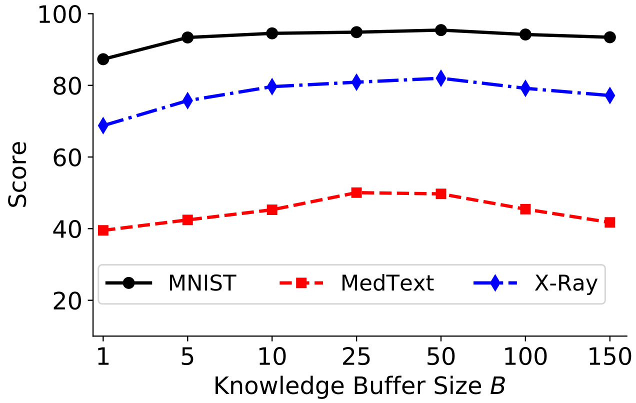

Supplementary Figure 6. The influence of the knowledge buffer size on PrivateKT. The model performance under different knowledge buffer sizes based on three datasets are illustrated in the figures. The results show that a moderate knowledge buffer size (e.g., 25) is optimal for PrivateKT.

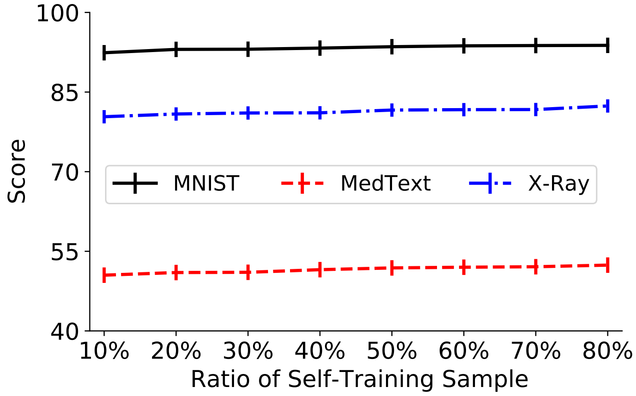

Supplementary Figure 7. The influence of the self-training sample size. The error bars represent the mean results with standard deviations. We evaluate the performance of PrivateKT under different self-training sample sizes (M). The results show that the self-training sample size is not a sensitive hyper-parameter for PrivateKT.

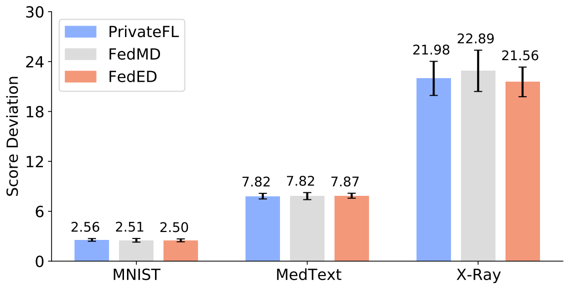

Supplementary Figure 8. The bias analysis of PrivateKT. The error bars represent the mean results with standard deviations. We evaluate the performance deviations of PrivateKT and other baseline knowledge transfer methods based on full public data (i.e., FedKD and FedMD). Results show that there is no significant difference between the performance deviations of PrivateKT and baseline methods, which reveals that the selected small data in PrivateKT does not lead to additional bias in the model training.

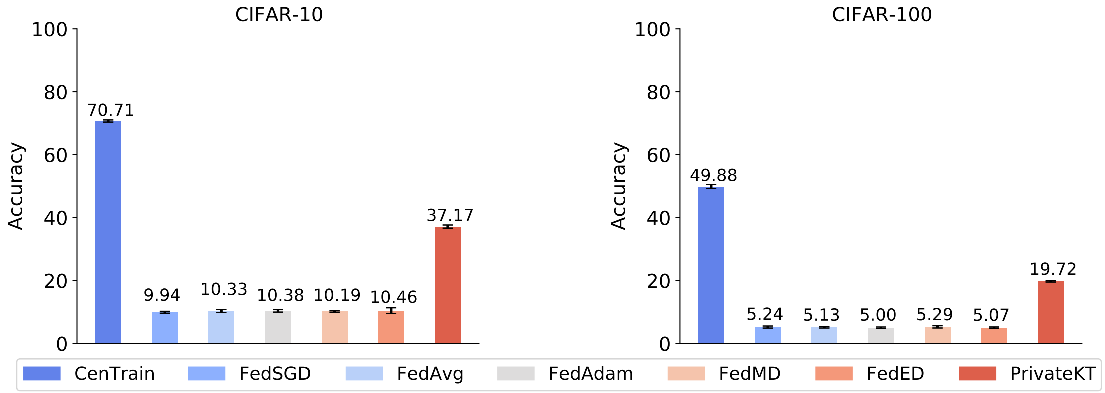

Supplementary Figure 9. Evaluation on larger FL benchmark datasets. The error bars represent the mean results with standard deviations. We conduct experiments to compare PrivateKT and baseline FL methods on CIFAR-10 and CIFAR-100. Results show that the main conclusions of our work still hold on these two datasets, demonstrating PrivateKT is applicable in most scenarios with small or moderately large data volumes.

Supplementary Tables

	MNIST	X-Ray
# training samples	48,000	4173
# test samples	10,000	624
# unlabeled public samples	12,000	1043
# classification categories	10	2
# local clients	80	53
# local training samples	600	80
degree of Non-IID data distribution α	0.5	0.5

Supplementary Table 1. Statistical information of the MNIST and X-Ray datasets. The two datasets have different sample sizes and numbers of classification categories. The number of partitioned local clients and the corresponding local sample sizes are also different for these two datasets.

# training samples	9,240	# test samples	2,888
# public samples	2,310	# classification categories	5
# local clients	31	# local training samples	300
avg. text length	179.94	degree of Non-IID data distribution α	0.5

Supplementary Table 2. Statistical information of the MedText dataset. The average text length means the average number of words in the medical records.

	MNIST	MedText	X-Ray
ratio of sampled clients in each round	50%	50%	50%
optimizer for local model training	SGD	Adam	SGD
learning rate for local model training	0.05	0.0001	0.05
size of knowledge transfer data K	2	2	2
privacy budget ε	5	5	5
size of knowledge buffer B	25	25	25
ratio of public data for self-training	10%	10%	10%
optimizer for global model fine-tuning	SGD	Adam	SGD
learning rate for global model fine-tuning	0.05	0.0001	0.05
dropout probability	0.2	0.2	0.2
knowledge transfer rounds	500	1000	50

Supplementary Table 3. The hyper-parameter settings of PrivateKT on three datasets. We set different optimizers and learning rates for model training on these three datasets.

	FedSGD	FedAvg	FedAdam	FedMD	FedED	PrivateKT
Computational Complexity of Client	$\mathcal{O}(Nd^3)$	$\mathcal{O}(Nd^3)$	$\mathcal{O}(Nd^3)$	$\mathcal{O}(Nd^3)$	$\mathcal{O}(Nd^3)$	$\mathcal{O}(Nd^3)$
Computational Complexity of Sever	$\mathcal{O}(Hd)$	$\mathcal{O}(Hd)$	$\mathcal{O}(Hd)$	$\mathcal{O}(Ed^3)$	$\mathcal{O}(Ed^3)$	$\mathcal{O}((B+M)d^3)$

Supplementary Table 4. Computational complexities of different methods. d denotes the number of model parameters, N denotes the number of local training samples, H denotes the number of participant clients in a single round, E denotes the number of unlabeled public samples, B denotes the knowledge buffer size and M denotes the number of self-training samples. The analyses show that, compared with baseline methods, PrivateKT only slightly improves the computing costs of the server and has comparable computing costs for the client.

Supplementary Algorithm Pseudo Code

Supplementary Algorithm 1 Class non-IID data distribution partition strategy

Setting the hyper-parameter α that controls the degree of data class distribution imbalance
Setting the size of local dataset n
Setting the number of classification categories C
Putting all available training data in the set $\mathscr{D}_a$
Calculating the number of local clients $H = \lfloor \frac{|\mathscr{D}_a|}{n} \rfloor$
for i in $1, 2, ..., H$ **do**
    Draw label distribution $\mathbf{p}^i \in \mathbb{R}^C$ from $Dir(\alpha \cdot \mathbf{1})$ for the i-th client
    Initialize an empty local dataset $\mathscr{D}_l^i$
    for j in $1, 2, ..., C$ **do**
        $n_j = \lfloor n \times \mathbf{p}_j^i \rfloor$
        Randomly sample n_j data belonging to the j-th category from $\mathscr{D}_a$
        Put sampled data in $\mathscr{D}_l^i$
    end for
    Update the available training dataset: $\mathscr{D}_a = \mathscr{D}_a - \mathscr{D}_l^i$
end for
return $\{\mathscr{D}_l^i | i = 1, 2, ..., H\}$

Supplementary Algorithm 2 Size non-IID data distribution partition strategy

Setting the hyper-parameter α that controls the degree of data size distribution imbalance
Putting all available training data in the set $\mathscr{D}_a$
Setting the number of the current local clients H to zero (i.e., $H = 0$)
while $|\mathscr{D}_a| > 0$ **do**
    Update the number of the current local clients: $H = H + 1$
    Draw local data size n from right-side Laplace Distribution $|La(0, \alpha)|$ for the H-th client
    Randomly sample n data from $\mathscr{D}_a$ to form local training data set $\mathscr{D}_l^H$
    Update the available training dataset: $\mathscr{D}_a = \mathscr{D}_a - \mathscr{D}_l^H$
end while
return $\{\mathscr{D}_l^i | i = 1, 2, ..., H\}$

Supplementary Algorithm 3 Mixed non-IID data distribution partition strategy

Setting the hyper-parameter α_c that controls the degree of data class distribution imbalance and hyper-parameter α_s that controls the degree of data size distribution imbalance
Setting the number of classification categories C
Putting all available training data in the set $\mathscr{D}_a$
Setting the number of the current local clients H to zero (i.e., $H = 0$)
while $|\mathscr{D}_a| > 0$ **do**
    Update the number of the current local clients: $H = H + 1$
    Draw local data size n from right-side Laplace Distribution $|La(0, \alpha_s)|$ for the H-th client
    Draw label distribution $\mathbf{p}^H \in \mathbb{R}^C$ from $Dir(\alpha_c \cdot \mathbf{1})$ for the H-th client
    Initialize an empty local dataset $\mathscr{D}_l^H$
    for j in $1, 2, ..., C$ **do**
        $n_j = \lfloor n \times \mathbf{p}_j^H \rfloor$
        Randomly sample n_j data belonging to the j-th category from $\mathscr{D}_a$
        Put sampled data in $\mathscr{D}_l^H$
    end for
    Update the available training dataset: $\mathscr{D}_a = \mathscr{D}_a - \mathscr{D}_l^H$
end while
return $\{\mathscr{D}_l^i | i = 1, 2, ..., H\}$
