# Supplementary material for: Differentially private knowledge transfer for federated learning
Source: Nat Commun. 2023 Jun 24;14:3785. doi: 10.1038/s41467-023-38794-x (PMC10290720; doi:10.1038/s41467-023-38794-x)
Supplement: Supplementary file 3 — Reporting Summary [file 41467_2023_38794_MOESM3_ESM.pdf]

nature portfolio

Corresponding author(s): Fangzhao Wu, Chuhan Wu, Yongfeng Huang

Last updated by author(s): May 7, 2023

Reporting Summary

Nature Portfolio wishes to improve the reproducibility of the work that we publish. This form provides structure for consistency and transparency in reporting. For further information on Nature Portfolio policies, see our Editorial Policies and the Editorial Policy Checklist.

Statistics

For all statistical analyses, confirm that the following items are present in the figure legend, table legend, main text, or Methods section.

n/a	Confirmed	
☒	☐	The exact sample size (*n*) for each experimental group/condition, given as a discrete number and unit of measurement
☐	☒	A statement on whether measurements were taken from distinct samples or whether the same sample was measured repeatedly
☐	☒	The statistical test(s) used AND whether they are one- or two-sided<br>*Only common tests should be described solely by name; describe more complex techniques in the Methods section.*
☒	☐	A description of all covariates tested
☐	☒	A description of any assumptions or corrections, such as tests of normality and adjustment for multiple comparisons
☐	☒	A full description of the statistical parameters including central tendency (e.g. means) or other basic estimates (e.g. regression coefficient) AND variation (e.g. standard deviation) or associated estimates of uncertainty (e.g. confidence intervals)
☐	☒	For null hypothesis testing, the test statistic (e.g. *F*, *t*, *r*) with confidence intervals, effect sizes, degrees of freedom and *P* value noted<br>*Give P values as exact values whenever suitable.*
☒	☐	For Bayesian analysis, information on the choice of priors and Markov chain Monte Carlo settings
☐	☒	For hierarchical and complex designs, identification of the appropriate level for tests and full reporting of outcomes
☒	☐	Estimates of effect sizes (e.g. Cohen's *d*, Pearson's *r*), indicating how they were calculated

Our web collection on statistics for biologists contains articles on many of the points above.

Software and code

Policy information about availability of computer code

Data collection	No software was used for data collection.
Data analysis	The PrivateKT framework was implemented by the Keras 2.2.4 library. The Python version is 3.6.9. Analysis was performed by numpy 1.19.5, scipy 1.5.2 . More details can be found in the manuscript methods section, supplementary materials, and the code repository https://github.com/taoqi98/PrivateKT.

For manuscripts utilizing custom algorithms or software that are central to the research but not yet described in published literature, software must be made available to editors and reviewers. We strongly encourage code deposition in a community repository (e.g. GitHub). See the Nature Portfolio guidelines for submitting code & software for further information.

Data

Policy information about availability of data

All manuscripts must include a data availability statement. This statement should provide the following information, where applicable:

- Accession codes, unique identifiers, or web links for publicly available datasets
- A description of any restrictions on data availability
- For clinical datasets or third party data, please ensure that the statement adheres to our policy

The datasets that supports the findings of this study are all publicly available ones, and the use of them in this work adheres to the licenses of these datasets. The MNIST dataset is available at http://yann.lecun.com/exdb/mnist/. The MedText dataset is available at https://www.kaggle.com/datasets/chaitanyakck/medical-text.

The X-Ray dataset is available at https://www.kaggle.com/datasets/paultimothymooney/chest-xray-pneumonia. The CIFAR-10 and CIFAR-100 datasets are available at https://www.cs.toronto.edu/~kriz/cifar.html.

Research involving human participants, their data, or biological material

Policy information about studies with human participants or human data. See also policy information about sex, gender (identity/presentation), and sexual orientation and race, ethnicity and racism.

Reporting on sex and gender	Our research does not involve human data.
Reporting on race, ethnicity, or other socially relevant groupings	Our research does not involve human data.
Population characteristics	Our research does not involve human data.
Recruitment	Our research does not involve human data.
Ethics oversight	Our research does not involve human data.

Note that full information on the approval of the study protocol must also be provided in the manuscript.

Field-specific reporting

Please select the one below that is the best fit for your research. If you are not sure, read the appropriate sections before making your selection.

☒ Life sciences ☐ Behavioural & social sciences ☐ Ecological, evolutionary & environmental sciences

For a reference copy of the document with all sections, see nature.com/documents/nr-reporting-summary-flat.pdf

Life sciences study design

All studies must disclose on these points even when the disclosure is negative.

Sample size	There are five datasets used in this work and all of them have thounds of samples.
Data exclusions	We conduct experiments on the raw collected dataset and do not perform any data exclusion operations.
Replication	The experimental results can be replicated by applying the algorithm to each sample in the test set, and can be further validated by conducting experiments on other data with similar formats.
Randomization	The experimental datasets were randomly divided into training, validation and test sets. Each sample was assigned to exactly one of these splits.
Blinding	When assigning samples randomly to different sets, researchers were blinded to the sample selection. Research team members were also blinded to the data collection process and all the identities of users and samples had been fully anonymized.

Reporting for specific materials, systems and methods

We require information from authors about some types of materials, experimental systems and methods used in many studies. Here, indicate whether each material, system or method listed is relevant to your study. If you are not sure if a list item applies to your research, read the appropriate section before selecting a response.

Materials & experimental systems

n/a	Involved in the study	
☒	☐	Antibodies
☒	☐	Eukaryotic cell lines
☒	☐	Palaeontology and archaeology
☒	☐	Animals and other organisms
☒	☐	Clinical data
☒	☐	Dual use research of concern
☒	☐	Plants

Methods

n/a	Involved in the study	
☒	☐	ChIP-seq
☒	☐	Flow cytometry
☒	☐	MRI-based neuroimaging
