# Supplementary material for: Differentially private knowledge transfer for federated learning
Source: Nat Commun. 2023 Jun 24;14:3785. doi: 10.1038/s41467-023-38794-x (PMC10290720; doi:10.1038/s41467-023-38794-x)
Supplement: Supplementary file 2 — Peer review file [file 41467_2023_38794_MOESM2_ESM.pdf]

nature portfolio

Peer Review File

Differentially Private Knowledge Transfer for Federated Learning

Open Access This file is licensed under a Creative Commons Attribution 4.0 International License, which permits use, sharing, adaptation, distribution and reproduction in any medium or format, as long as you give appropriate credit to the original author(s) and the source, provide a link to the Creative Commons license, and indicate if changes were made. In the cases where the authors are anonymous, such as is the case for the reports of anonymous peer reviewers, author attribution should be to 'Anonymous Referee' followed by a clear attribution to the source work. The images or other third party material in this file are included in the article's Creative Commons license, unless indicated otherwise in a credit line to the material. If material is not included in the article's Creative Commons license and your intended use is not permitted by statutory regulation or exceeds the permitted use, you will need to obtain permission directly from the copyright holder. To view a copy of this license, visit http://creativecommons.org/licenses/by/4.0/.

Editorial Note: This manuscript has been previously reviewed at another journal that is not operating a transparent peer review scheme. This document only contains reviewer comments and rebuttal letters for versions considered at Nature Communications.

Response Letter

We thank the reviewers and editors for the insightful comments and great suggestions. We have carefully revised our paper according to the previous comments, and there is no new comment from reviewers in this revision round. We highly appreciate the meticulous reading, insightful comments, and detailed feedback provided by the editorial team and anonymous reviewers. They are really helpful in improving the quality of our work and meeting the requirements of publication.
